# Effects of Context on Visuomotor Interference Depends on the Perspective of Observed Actions

**DOI:** 10.1371/journal.pone.0053248

**Published:** 2013-01-03

**Authors:** Marta Bortoletto, Jason B. Mattingley, Ross Cunnington

**Affiliations:** 1 Cognitive Neuroscience Unit, IRCCS San Giovanni di Dio Fatebenefratelli, Brescia, Italy; 2 School of Psychology and Queensland Brain Institute, The University of Queensland, St Lucia, Queensland, Australia; University of Bologna, Italy

## Abstract

Visuomotor interference occurs when the execution of an action is facilitated by the concurrent observation of the same action and hindered by the concurrent observation of a different action. There is evidence that visuomotor interference can be modulated top-down by higher cognitive functions, depending on whether own performed actions or observed actions are selectively attended. Here, we studied whether these effects of cognitive context on visuomotor interference are also dependent on the point-of-view of the observed action. We employed a delayed go/no-go task known to induce visuomotor interference. Static images of hand gestures in either egocentric or allocentric perspective were presented as “go” stimuli after participants were pre-cued to prepare either a matching (congruent) or non-matching (incongruent) action. Participants performed this task in two different cognitive contexts: In one, they focused on the *visual image* of the hand gesture shown as the go stimulus (image context), whereas in the other they focused on the hand gesture they *performed* (action context). We analyzed reaction times to initiate the prepared action upon presentation of the gesture image and found evidence of visuomotor interference in both contexts and for both perspectives. Strikingly, results show that the effect of cognitive context on visuomotor interference also depends on the perspective of observed actions. When focusing on own-actions, visuomotor interference was significantly less for gesture images in allocentric perspective than in egocentric perspective; when focusing on observed actions, visuomotor interference was present regardless of the perspective of the gesture image. Overall these data suggest that visuomotor interference may be modulated by higher cognitive processes, so that when we are specifically attending to our own actions, images depicting others’ actions (allocentric perspective) have much less interference on our own actions.

## Introduction

It is well established that seeing someone else performing an action facilitates the execution of the same action and hinders the execution of a different action. This priming effect has been referred to as automatic imitation [1], visuomotor priming [2], [3], or motor mimicry [4], and has been shown by recording reaction times (RTs) to initiate a movement in response to the presentation of images of hand gestures that are compatible or incompatible with the response. We will use the term “visuomotor interference” to highlight that this phenomenon derives from a strong and direct interaction between the visual system and the motor system, which has its basis in the overlap of neural activity within parietal and premotor cortical areas that are activated during both action execution and action observation [5]–[7].

The interaction between the visual system and the motor system has been proposed as a crucial mechanism for a variety of cognitive skills. First, it may underlie action understanding by activating the motor representation corresponding to the observed action and so linking the visual information with the internal motor repertoire [8] or by activating predictive models [9], [10]. Moreover, it may support a direct matching process to activate the representation of the observed action in the motor system during imitation [11]. Last, it may reflect the influence of predicted sensory consequences of actions in motor control [12]. Indeed, according to the ideomotor theory of action [13] and to the theory of event coding [14], [15], representations of actions include their perceptual consequences that are activated in the preparatory phases of movements to select and guide voluntary actions [For a review on the main theories on action-perception interaction see [16]].

There is evidence that the link between the visual system and the motor system may work in two directions. Neuroimaging studies of cross-modal repetition suppression have shown that when repeated visual presentation of actions is followed by the execution of the same action, neural activity is suppressed in the inferior frontal gyrus [17] and in the inferior parietal lobe [18], [19]. Vice-versa, repeated action execution suppresses the cortical activity evoked by the subsequent observation of the same action in the inferior frontal gyrus [17]. Moreover, by using visual-evoked potentials and transcranial magnetic stimulation (TMS), recent studies have shown that motor plans can influence the perceptual processing of observed actions when hand gestures are observed concurrently with movement preparation [20]–[22]. Visuomotor interference effects may therefore derive from bidirectional links between visual and motor systems, involving two different priming effects: visual-to-motor priming in which the visual system influences movement representations in the motor system, and motor-to-visual priming in which the motor system influences perceptual processing in the visual system.

A crucial question is whether visuomotor interference is an automatic process or can be top-down modulated to adapt to different cognitive situations. Indeed, there are many examples in which it would be highly detrimental for our own behavior to be prone to visuomotor interference. A basketball player taking free throw, focusing on their own action, does not want their throw to be influenced by observed actions of others (visual-to-motor priming). Likewise, a boxer needs to maintain fast and accurate perceptual processing of the movements of his opponent that is not influenced by the actions he is planning himself (motor-to-visual priming). Previous studies suggest that visuomotor interference is modulated by higher cognitive functions such as attention [23], [24] and social interaction [25], [26]. Directing attention to an observed action [4], [23], [24], or to specific action-related features of a stimulus [27], [28], increases visuomotor interference and influences cortical activity associated with action observation [18], [29].

Visuomotor interference is also influenced by task instructions or demands that shape the cognitive context of the task. For example, whether identical actions or complementary actions (i.e. non-identical but goal-related actions) are facilitated during action observation is determined in a dynamic, context-dependent fashion [30]. Participants’ intentions, either to understand an action or to identify physical features, is associated with distinct patterns of cortical activation during action observation [31]. Interestingly, cognitive context appears to change the strength and direction of visuomotor interactions.

Here, we aimed to further investigate the effect of cognitive context on visuomotor interference, when actions are perceived from different point-of-views: an egocentric perspective (the observer sees the action as if it was performed by himself/herself) and an allocentric perspective (as if the observer faces someone else performing the action). Previous studies have shown that the perspective from which actions are observed modulates the interaction between the visual system and the motor system and the cortical activity associated with action observation [3], [32]–[34]. In line with the theory of event coding [14], [15], images of actions presented in the egocentric perspective may trigger stronger visuomotor interference than images in the allocentric perspective [32], [35]. Nevertheless, it is possible that the effect of perspective varies with the cognitive context, as described below. Considering that when we perform an action, the visual outcome of our motor plan is always perceived in egocentric perspective, cognitive contexts in which our own actions are more relevant may facilitate the representation of the action as if performed by the observer (i.e. in the egocentric perspective). Therefore, in these contexts visuomotor interference should be stronger for observation of actions in the egocentric perspective than in the allocentric perspective. Conversely, others’ actions are perceived in many different ways, generally allocentric perspective but also in egocentric perspective. Therefore, cognitive contexts in which others’ actions are more relevant, should be associated with stronger visuomotor interference for the allocentric perspective or equivalent effects for the egocentric and the allocentric perspectives.

By employing a delayed go/no-go task involving images of hand gestures and measuring reactions times to the presentation of these images, visuomotor interference should be apparent as a slowing of reaction times to initiate movements in response to incongruent gestures compared to congruent gestures. We hypothesized that the cognitive context would influence visuomotor interference effects according to the point-of-view of the observed action.

## Methods

### Participants

Thirty right-handed healthy volunteers (15 females, aged between 18 and 27, mean age: 19.2) gave their written informed consent to participate in the study, and the study was approved by the Medical Research Ethics Committee of The University of Queensland.

### Procedure

Participants were comfortably seated in a dimly-lit room, facing an LCD monitor placed 70 cm in front of them. Their hands were resting on two response boxes placed in a comfortable position on a table. The right arm and the corresponding response box were hidden in a cardboard box so that participants could not see the gestures made with their right hand.

Before beginning the experiment, all participants performed a short training to familiarize with the actions employed in the task. One of four target words representing the four required gestures (‘OK’, ‘Peace’, ‘Thumbs-up’, and ‘Point’) was displayed for 1s, followed by the corresponding hand gesture image displayed either in egocentric or in allocentric perspective. Participants silently read the word and performed the hand gesture with their right hand. Each gesture was repeated eight times.

**Figure 1:**
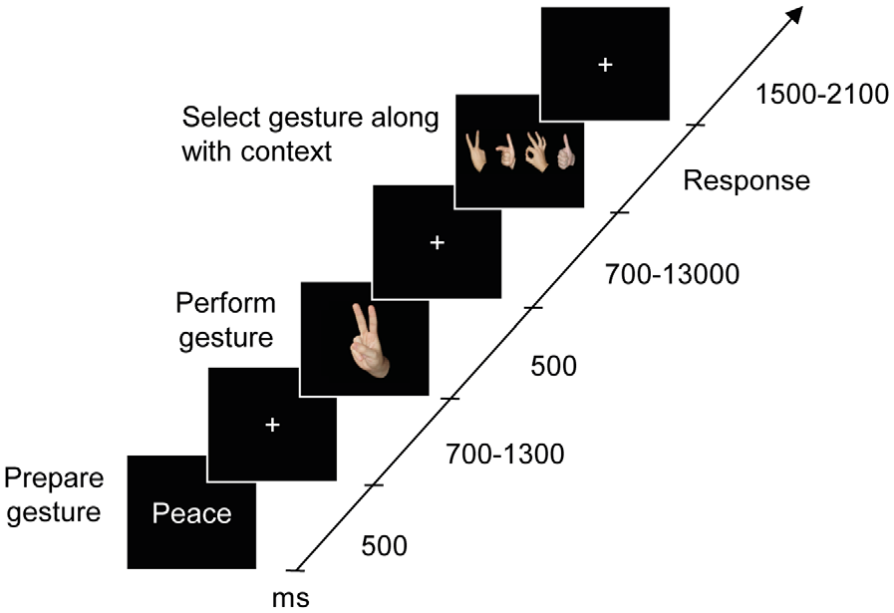
Time course of a typical trial of the go/no-go task. In each trial, an initial word cue indicated one of four possible hand gestures (‘OK’, ‘Peace’, ‘Thumbs-Up’, ‘Point’). Participants prepared and performed the cued gesture with their right hand as quickly as possible in response to ‘Go’ cues (67% of trials), and withheld responses to ‘No-go’ cues (33%). ‘Go’ cues consisted of a static image of a hand gesture that either matched (congruent) or did not match (incongruent) the cued and executed action, presented either in the egocentric perspective or in the allocentric perspective. Reaction times (RTs) were measured. After performing the action, participants were required to report either the action they had just performed (action context), or the action they had just seen (image context) and to select the correct response among the four possible gestures.

The experiment consisted of a delayed go/no-go task (Figure 1). In each trial, participants were required to prepare one of four possible actions, as indicated by a word cue, and to perform the action with their right hand as quickly as possible upon the presentation of the go stimulus. Participants started their responses by releasing a resting-position button so that RTs to movement initiation were recorded. The actions performed by participants were monitored by the experimenter via an infrared camera mounted inside the occluding box around the participant’s right hand. Go stimuli were photographs of gestures performed with the right hand, in the egocentric or allocentric perspective, which either matched the prepared action (congruent trials), or were one of the other three non-matching gestures (incongruent trials). Gesture images were presented centrally at fixation, and size of within about 10 degrees visual angle. To avoid a disparity in the frequency of presentation between congruent and incongruent gestures, each gesture was paired with one of the other gestures and in incongruent trials only the paired incongruent gesture was used. All possible pairings of gestures were used, with different pairings for each participant, counterbalanced between participants. The no-go stimulus was indicated by a stop hand gesture and was presented in one third of the trials.

To manipulate the cognitive context, participants were instructed at the start of each block that they would be required to report either the hand action they had performed (action context) or the hand action they had seen (image context). Following a variable delay of 700–1300 ms after the presentation of the go/no-go stimulus, participants were presented an horizontal array of all four gestures and were asked to select the appropriate gesture by pressing the corresponding button on a 4-button response-pad with their left hand. In the image context, participants indicated which action they had seen, while in the action context they indicated which action they had performed. For these images, the target hand gesture and the other gestures were depicted randomly either in the egocentric or allocentric perspective, irrespective of the perspective of the go stimulus.

At the beginning of the experiment, participants performed two practice blocks of 24 trials, one for each context. In these blocks, the selection of the action to be remembered was followed by feedback on responses. Then, for experiment trials, participants performed 5 blocks of 52 trials alternately for each cognitive context, with the order counterbalanced between participants. The first four trials of each block were discarded from analyses to avoid possible task switching effects. These trials were pseudorandomly selected in each block so that overall each block included all possible trials and did not affect the counterbalancing of the analyzed trials. A total of 480 trials were analyzed, consisting of 40 trials for each condition (2 contexts – action and image, by 2 perspectives – egocentric and allocentric, by 2 congruency – congruent and incongruent), and 80 no-go trials for each context.

### Statistical Analyses

We measured reaction times (RTs) to the go stimulus, i.e. the time to initiate the prepared action after the presentation of the go stimulus. Responses were considered errors and were excluded from analyses if RTs were faster than 120 ms (anticipations) and if RTs were more than three standard deviations above the average (misses).

In order to assess overall visuomotor interference effects for each condition, paired t-tests were used to compare differences in RTs between congruent and incongruent gestures, for each context and for each perspective. In order to test differences in visuomotor interference between contexts and perspectives, the differences in RTs between congruent and incongruent gestures were calculated and values were analyzed in a 2-way repeated-measures ANOVA that included factors of Context (Action context, Image context) and Perspective of hand images (Egocentric, Allocentric). Fisher’s LSD correction for multiple comparisons was used for post-hoc analyses.

Several measures allowed us to confirm that participants performed the task according to instructions. First, we measured errors at the presentation of the gesture images by counting numbers of anticipations, misses and wrongly performed actions for the go cue, as well as false alarms to the no-go cue. We also counted errors for wrong responses when participants selected which action they had seen (in the image context) and which action they had performed (in the action context). Two participants performed more than 30% false alarms at the presentation of the no-go stimulus and were excluded from further analyses.

## **Results**

In line with previous studies, initiating an action was facilitated by the congruency between the observed action and the prepared action. As shown in Table 1, this effect was statistically significant when gesture images were presented both in egocentric and allocentric perspective and in both action and image contexts.

**Table 1:** Summary of interference effects in milliseconds.

	Congruentgesture	Incongruentgesture	Difference	T-test
*Action context*				
Egocentric	551 (148)	593 (178)	42	3.23*
Allocentric	564 (155)	585 (167)	21	3.01*
*Image context*				
Egocentric	557 (153)	597 (173)	40	3.53*
Allocentric	551 (150)	597 (169)	46	5.51*

RTs for congruent gestures, RTs for incongruent gestures, difference and the T-test value. Numbers in brackets indicate standard deviation. Asterisks indicate p<.01.

**Figure 2:**
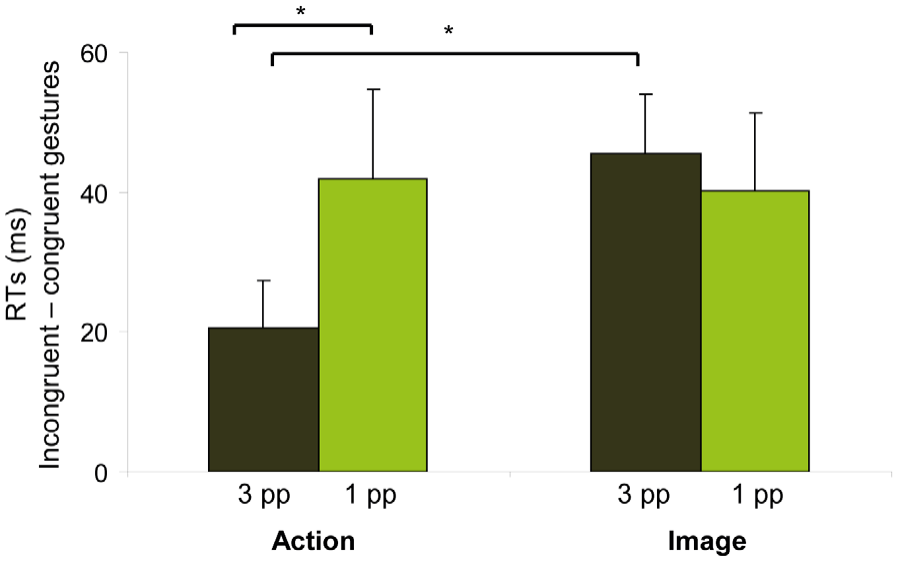
Effects of context and perspective on visuomotor interference. Values indicate the difference in reaction times (RTs) between incongruent and congruent gestures (congruency effect). The congruency effect for the allocentric perspective was reduced compared to egocentric perspective in the action context and was reduced in the action context compared with the image context. Asterisks indicate p<.05.

Crucially, comparisons across conditions revealed that the influence of cognitive context on visuomotor interference depended on perspective of the observed actions. Indeed, the visuomotor interference effect was modulated by perspective and cognitive context [Context x Perspective interaction, F(1, 27) = 4.31, p<.05] (Figure 2). Paired t-tests showed that, in the action context, the congruency effect was significantly less for allocentric (3 pp) compared with egocentric (1 pp) perspective gesture images [t(27) = 2.27, p<.05]; however, in the image context, the congruency effect was present regardless of the perspective of the gesture image [t(27) = .56, p>.05]. Similarly, congruency effects were also significantly less for allocentric (3 pp) perspective images in the action context compared with the image context [t(27) = 2.88, p<.05], while congruency effects for egocentric perspective images did not differ with context [t(27) = .12, p>.05]. In other words, the interaction between cognitive context and perspective was obtained because the visuomotor interference effect was reduced for actions observed in the allocentric perspective in the action context. This result suggests that, when participants specifically attend to the action they perform, the representation of the action in third-person allocentric perspective may be suppressed and therefore cause less visuomotor interference.

Participants made very few errors across conditions and contexts. The percentage of errors at the presentation of the gesture image was very low, with less than 3% for anticipations, misses and wrongly performed actions combined. The percentage of false alarms at the presentation of the no-go stimulus was significantly greater in the image context (6.0%) than in the action context (5.0%), t(27) = 3.75, p<.05, indicating that when the performed action was more relevant, participants had better control over their movements. Less than 5% errors were made in the selection of the gesture to be remembered in both contexts. Nevertheless, participants made significantly more errors when they had to select the action they had seen (image context = 4.9%) compared with when they had to select the action they had performed (action context = 2.3%), t(27) = 2.98, p<.05. This result suggests that remembering the observed action may be more difficult than remembering the performed action.

## Discussion

Our results show that visuomotor interference during action observation and its relationship with the cognitive context can change depending on the point-of-view of the observer. Overall these data suggest that visuomotor interference may be modulated by higher cognitive processes, so that when we are specifically attending to our own actions, others’ actions (i.e. actions observed in the allocentric perspective) have much less interference on our own actions.

Our study extends on previous findings by showing that the effect of perspective on visuomotor interference depends on the contingent cognitive situation and highlights the relevance of focusing on our own or others’ actions as a critical factor. Studies on visuomotor interference have generally employed paradigms in which the processing of observed actions was task-irrelevant, as in our action context. Such studies generally find larger effects for egocentric than allocentric perspective. Indeed, they have found that the facilitation of cortico-spinal excitability during action observation that has previously been shown in TMS studies of motor-evoked potentials (MEPs) [36] is stronger for actions observed from the egocentric perspective than for actions observed from the allocentric perspective [37], [38]. These data have been interpreted according to the theory of event coding [14], [15] suggesting that visuomotor interference effects are stronger for the egocentric perspective because of the higher dimensional overlap of the observed and executed action [39]. Accordingly, our results also show that visuomotor interference is stronger for the egocentric perspective than for the allocentric perspective in the action context.

However, a different pattern was found in the image context, in which actions observed from either egocentric or allocentric perspectives caused similar visuomotor interference. In line with our results, a few studies have reported no differences in the activity within the action-observation network when egocentric and allocentric perspectives were compared [40], [41]. Moreover, it has previously been found that the effect of perspective varies across paradigms [3], [38] so that in some circumstances, actions observed in allocentric perspective can induce strong interference between the visual system and the motor system. For example, in Vogt et al [3], the egocentric perspective was associated with stronger visuomotor interference only when hand gestures (presented as primes) were preceded by a fixation-stimulus depicting a hand in a resting position. When the fixation-stimulus was a dot, there was stronger visuomotor interference for the allocentric perspective. Therefore, the relevance of the observed action, or the context, may modulate the pattern of the perspective effects.

Visuomotor interference effects are based on the correspondence between the configural properties of the observed action and the configural properties of the performed action. Spatial compatibility, i.e. the facilitation of performing actions that share spatial features with the observed actions, has been shown to induce effects similar to visuomotor interference effects and is considered one of the major confounds in the study of these effects [42]–[44]. Indeed, gesture images that are congruent with a performed action share configural features and potentially also spatial features. To control for spatial compatibility effects, we carefully constructed the images so that they did not have overall more critical features on the left versus right side, and they had roughly equal pixels left and right. Therefore it is unlikely that spatial compatibility can explain our results.

An important factor regarding the effect of perspective is that observing actions from different view-points may lead to different attributions of the agent’s identity, distinguishing between others’ actions and own actions. Indeed, beliefs regarding agency have been suggested to be a critical factor that modulates visuomotor interaction during action observation [45]–[48]. By manipulating the sense of ownership through the rubber-hand illusion, Schutz-Bosbach et al [45] have shown that motor facilitation depends on attributing the observed action to self or to others. Likewise, in our study the perspective from which actions were observed may have led to differences in the way agency of the performing hand was represented. The allocentric perspective may have been associated with others’ actions, as we hardly ever see our own actions from this perspective; the egocentric perspective may be more easily associated with our own actions, but can also represent actions performed by others. As a consequence, when our own actions were more relevant for the task, the processing of others’ actions may have been suppressed leading to little visuomotor interference of the allocentric action images. In contrast, both egocentric and allocentric perspectives may represent actions performed by others; therefore, they similarly induced visuomotor interference effects in the context in which the perceived action was more relevant.

We interpret our results as a reduction of visuomotor interference for actions observed in the allocentric perspective when performed actions are most relevant. Indeed allocentric perspective in the action context induces less interference compared with all other conditions of the task (egocentric perspective in the action context, and both perspectives in the image context). Nevertheless, other mechanisms are possible. Namely, attention to the observed actions, as in the image context, could increase the effect of observed actions on the motor system and therefore increase visuomotor interference. This effect may be evident only for actions observed in the allocentric perspective, because visuomotor interference for egocentric actions is stronger and may not further increase.

Interestingly, this interaction between cognitive context and perspective may suggest that the link between the visual system and the motor system is top-down controlled. Nevertheless, according to a recent model of automatic imitation [1], the modulation of visuomotor interference could occur at different stages. First, changing the relevance of observed action may modulate the sensory input into the visuomotor transformation. Second, the context modulation may change the visuomotor transformation itself. Finally, changing the relevance of performed actions may modulate the motor output after the sensory motor transformation. Although at this stage it is not possible to disentangle these possibilities, here we suggest that a possible mechanism underlying this effect is that context can change the direction of visuomotor interactions. The present data are in line with this hypothesis. Although reaction times are hardly sensitive to differences in the direction of visuomotor interference, as they reflect a combination of perceptual and motor planning processes, the study of the perspective from which an action is observed may provide insights into these mechanisms. Indeed, perspective can modulate the cortical activity associated with cross-modal action representations, i.e. the analogous coding of action across visual and motor domains [49]. As explained by Vogt and coworkers [3], motor-to-visual priming and visual-to-motor priming would predict different effects of perspective. Motor-to-visual priming predicts stronger effects in the egocentric perspective than in the allocentric perspective because the representation of our own action consequences is likely built on the sensory feedback of our own actions. In contrast, visual-to-motor priming would predict equivalent effects between perspectives, or perhaps even stronger effects in the allocentric perspective. According to this model, stronger visuomotor interference for the egocentric perspective in the action context may reflect stronger motor-to-visual priming. In contrast, equal visuomotor interference effects of the egocentric and allocentric perspective in the image context may derive from visual-to-motor priming. Therefore, depending on the context, the visual system and the motor system may interact in two different directions. Nevertheless, this conclusion can only be speculative at this stage and further studies are needed to support it, for example by measuring in the same paradigm the cortical activity associated with action observation and the cortical activity associated with action preparation in different cognitive contexts.

It should be noted that the distinction between visual-to-motor priming and motor-to-visual priming does not necessarily mean that the information flow is unidirectional, rather it indicates which information is influenced by the concurrent processing of observed actions and motor plans. Our data suggest that visuomotor interactions may be top-down controlled, through executive functions, to preserve the activated motor plans during social interaction. This is supported by evidence that, when we specifically attend to our own actions, interference effects from observing others’ actions are reduced. Therefore, in this respect, our results are consistent with contemporary theories of predictive models [9], [10] that are based on recurrent interactions between systems.

Visuomotor interference reveals that there is a mechanism by which motor representations may be activated through visual stimulation, as supported by the findings that action observation induces neural activity in the motor system and activates cortical motor circuits in a similar way to action execution [36], [50]. Our data contribute substantially to understanding the role of perspective and context on visuomotor interactions and for understanding the parameters that are most effective for visual stimulation of the motor system during action observation. Our results suggest that when action performance is relevant to the contingent situation, it may be more effective to present visual stimuli representing actions in the egocentric rather than in the allocentric perspective. Given that action observation is increasingly considered for observational learning and motor neuro-rehabilitation [51]–[53], our results are relevant for the development of clinical trials, particularly for the way actions are observed during treatment.

This study highlights that the perspective from which actions are observed is not trivial, but rather can reveal important insights into the way the visual system and the motor system interact.
